# Impact of Superantigen-Producing Bacteria on T Cells from Tonsillar Hyperplasia

**DOI:** 10.3390/pathogens8030090

**Published:** 2019-06-27

**Authors:** Fiona J Radcliff, Sharon Waldvogel-Thurlow, Fiona Clow, Murali Mahadevan, James Johnston, Gen Li, Thomas Proft, Richard G Douglas, John D Fraser

**Affiliations:** 1 Department of Molecular Medicine and Pathology, University of Auckland, Auckland 1023, New Zealand; 2 Department of Surgery, University of Auckland, Auckland 1023, New Zealand

**Keywords:** superantigen, *Staphylococcus aureus*, *Streptococcus pyogenes*, Group A Streptococcus, TCR Vβ, mucosal-associated invariant T cells, recurrent tonsillitis, obstructive sleep apnea, tonsillar hyperplasia

## Abstract

*Staphylococcus aureus* and Group A Streptococcus (GAS) are common occupants of the tonsils and many strains produce potent exotoxins (mitogens) that directly target T cells, which could be a driver for tonsillar hyperplasia. Tonsil tissues from 41 patients were tested for these bacteria in conjunction with profiling of B and T cells by flow cytometry. *S. aureus* and GAS were detected in tonsil tissue from 44% and 7%, respectively, of patients by bacteriological culture; immuno-histology showed bacteria in close proximity to both B and T lymphocytes. The presence of tonsillar *S. aureus* did not alter B or T cell populations, whereas peripheral blood mucosal-associated invariant T (MAIT) cells were significantly increased in *S. aureus* culture positive individuals (*p* < 0.006). Alterations of tonsil CD4^+^ TCR Vβ family members relative to peripheral blood were evident in 29 patients. Three patients had strong TCR Vβ skewing indicative of recent exposure to superantigens, their tonsils contained mitogenic bacteria, and supernatants from these bacteria were used to partially recapitulate the skewing profile in vitro, supporting the notion that superantigens can target tonsillar T cells in situ. Tonsils are a reservoir for superantigen-producing bacteria with the capacity to alter the composition and function of key immune cells.

## 1. Introduction

A diverse range of microbes encompassing both commensal and pathogenic organisms have been isolated from human tonsils. There is ongoing interest in the flora associated with these tissues because enlargement of the tonsils (tonsillar hyperplasia) is a common indication for surgery. The requirement for surgery can arise either due to tonsillar hyperplasia causing conditions such as obstructive sleep apnea (OSA) or from recurrent tonsillitis (RT), which is caused by repeated infections. A tonsillectomy is one of the most frequent surgical procedures performed on children, with a growing proportion now performed to treat sleep apnea disorders such as OSA [1]. There are significant direct medical costs as well as an indirect economic burden associated with tonsillar hyperplasia [2,3]. Healthcare expenditure for treatment of acute and chronic RT in pediatric patients from the United States was estimated to be in excess of US $1.355 billion in 2015 [3]. Of particular concern is the high usage of antibiotics to treat these conditions.

Numerous microbial drivers encompassing viruses, bacteria, and fungi have been implicated in RT, with Group A Streptococci (GAS) considered to be the most common bacterial cause of RT in children [4]. Additional pathogenic microbes are commonly isolated from infected tonsils including *Staphylococcus aureus*, *Hemophilus influenzae,* and *Streptococcus pneumoniae* [5,6,7]. Some of these microbes are more frequently recovered from within tissue, rather than the tonsil surface, suggesting their role in tonsillar disease may be underestimated [5]. Both *S. aureus* and GAS can sequester in intracellular locations [8,9], or produce biofilms [10], which may allow these organisms to persist by limiting exposure to antibiotics and the host immune response.

*S. aureus* and GAS have a significant arsenal of virulence factors designed to disarm the host immune response and contribute to invasive disease [11,12]. Of particular interest in the context of tonsillar hyperplasia are superantigens (SAgs), potent exotoxins that cross-link T cells and antigen-presenting cells in a non-antigen restricted manner resulting in T cell activation, expansion, and inflammatory cytokine production [13,14]. Multiple serologically distinct SAgs have been identified from these bacteria, but possession of specific *sag* genes varies between isolates. Each SAg has specificity for one or more TCR Vβ family members, resulting in selective expansion, deletion, or anergy of targeted TCR Vβ subsets [14]. SAgs are best known for their role in life-threatening conditions such as toxic shock syndrome but are also linked to the pathogenesis of chronic or recurring conditions [15]. Studies using humanized MHC class II transgenic mice suggest SAgs are important for persistent colonization of the nasopharynx [16,17,18] as well as contributing to the severity of systemic disease [19,20]. 

Clinical studies have primarily focused on the impact of SAgs on the distribution of TCR Vβ family members and this information has been used to link SAg exposure to a range of clinical conditions including chronic rhinosinusitis [21], atopic dermatitis [22,23], and recurrent tonsillitis [24]. However, there is mounting evidence that SAgs impact on additional cell populations either directly or as a down-stream consequence of T-cell targeting [25,26]. Notably, SAgs can target and disarm innate lymphoid cells such as invariant natural killer T (*i*NKT) cells [27] and mucosal-associated invariant T (MAIT) cells [28] by cross-linking TCR Vβ on these cells with MHC class II on antigen-presenting cells (APCs). MAIT cells recognize selected microbial metabolites presented by major histocompatibility complex-related protein 1 (MR1) [29], including products from *S. aureus* [30,31] and GAS [32], forming a key component of the early response to these and other pathogenic microbes. Of direct relevance to studies of tonsillar disease, exposure of tonsillar cells to the common and potent Streptococcal pyogenic exotoxin A (SpeA) has been shown to have a negative impact on T follicular helper (TfH) cell activation and B cell viability, leading to significantly reduced production of Ig [33]. An in-depth examination of GAS RT patients indicated that many of these individuals have an HLA-linked susceptibility to SpeA, culminating in reduced numbers of TfH cells, smaller germinal centers in the tonsils, and a reduced antibody response to SpeA when compared to a matched cohort of OSA patients [34]. 

We have previously shown that *S. aureus* is common in tonsillar tissue, many isolates have the capacity to produce potent SAgs, and that the presence of *S. aureus* is frequently associated with perturbed TCR Vβ populations [24]. This study extends these observations in a new cohort of patients, using additional techniques to confirm the location of *S. aureus* or GAS within tonsil tissues and a comparison of matched tonsil and peripheral blood TCR Vβ profiles to verify local skewing of T cells. This study confirms that SAgs interact with immune cells in the tonsils, which may contribute to persistence or susceptibility to infection with *S. aureus* or GAS.

## 2. Results

### 2.1. Patient Demographics

Specimens from 41 donors with a median age of four years were examined, incorporating 23 tonsils removed to treat RT and 18 to alleviate symptoms associated with tonsillar hyperplasia, primarily obstructive sleep apnea (Table 1). There were no significant differences in gender, age, tonsil weight, leucocyte counts, or aerobic bacterial burden between these two groups of patients (Table 1). Thirty-two (78%) of patients took penicillin-based or broad-spectrum antibiotics in the three months leading up to surgery, 13 (32%) less than one month prior to surgery, with similar usage observed between the two patient groups.

### 2.2. Estimation of Bacterial Load

High numbers of bacteria were detected in tonsillar tissue after aerobic culture, typically exceeding 10^6^ CFU/g, irrespective of the reason for surgery (Table 1). *S. aureus* was cultured from homogenized tissue or from both tissue and a surface swab in 18 (44%) of individuals, including 5 RT and 13 tonsillar hyperplasia patients (Table 2). The quantity of *S. aureus* enumerated by culture was variable, ranging from occasional colonies to near-pure cultures (median = 13 × 10^4^ CFU/g, range = 0.023 − 430 × 10^4^ CFU/g). *S. pyogenes* was isolated from three RT patients (7%), two isolates were identified as *emm* 89 and one as *emm* 28. Quantification of these isolates by culture was similarly variable, ranging from 0.35, 55, and 128 × 10^4^ CFU/g. *Emm* typing of putative GAS isolates identified Group G Streptococci (GGS) StG62647, StG245, and Stg6792 in tonsil tissue from four patients and Group C Streptococcus (GGS) (StC47A) in a single individual.

### 2.3. Detection of S. aureus and GAS in Tonsil Tissue

**Figure 1:**
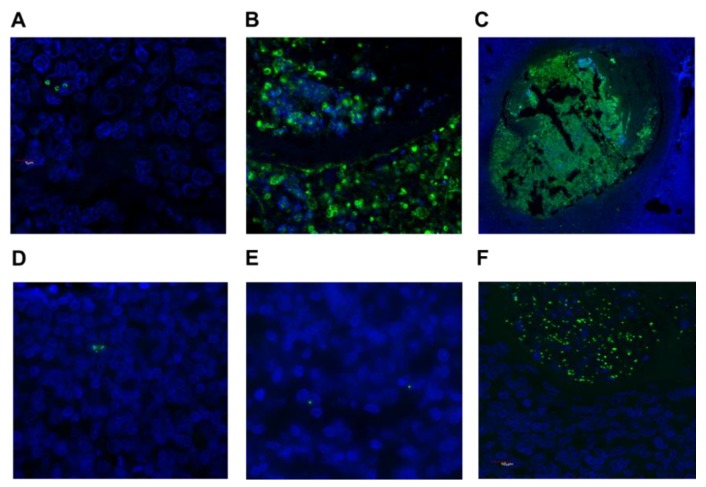
*S. aureus* and Group A Streptococcus (GAS) are located within tonsillar tissue. Bacteria were detected in paraffin-embedded tonsil tissue with antibodies specific for *S. aureus* or GAS (green), combined with a nuclear stain (DAPI, blue). Examples include *S. aureus* sparsely distributed in tonsillar tissue at 100× (**A**), clusters of bacteria at 100× (**B**) and at 10× (**C**); for GAS, occasional cocci are shown (**D**,**E**) and a large cluster of bacteria (**F**), all at 100× magnification.

Tissue sections from all 41 patients were labelled with antibodies targeting *S. aureus* or GAS and examined by fluorescence microscopy. This approach detected *S. aureus* in 15 patients and GAS in 3 patients (Table 2). Detection of bacteria by immuno-histology ranged from very occasional colonies (Figure 1A), small localized groups of colonies (Figure 1B), through to substantial clusters of bacteria (Figure 1C). There were discrepancies between *S. aureus* culture versus immuno-histology results (Table 2). Eight individuals were positive for *S. aureus* by both methods, whereas combining data from both techniques indicated that *S. aureus* was more common in the tonsils (25 individuals, 61%) than suggested by culture results alone. In line with this observation, scanning sequential sections through entire tonsils clearly demonstrates that bacteria are not evenly distributed throughout the tissue [35], highlighting the limitations of sampling small pieces from what can be sizeable tissues. GAS was detected in the three individuals that were culture positive for this bacterium (Figure 1D–F). 

### 2.4. Superantigen Profiling of Tonsillar S. aureus Isolates

**Figure 2:**
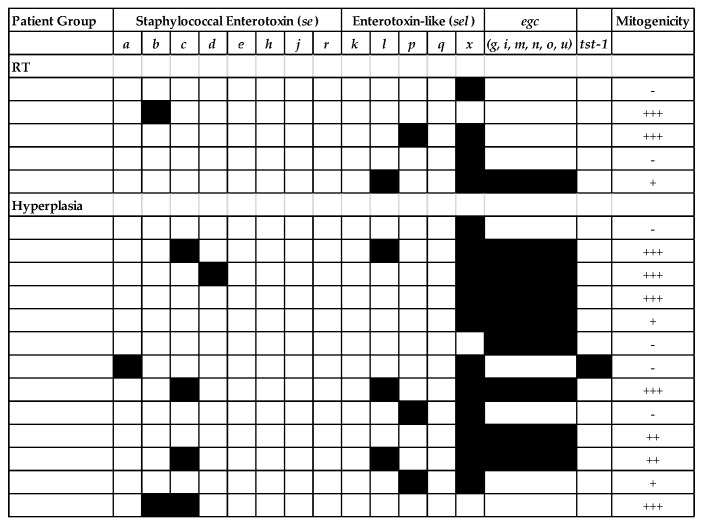
Presence of *sag* genes and mitogenicity of tonsil *S. aureus* isolates. A representative *S. aureus* isolate from each culture positive patient was tested for the presence of genes encoding for common Staphylococcal superantigens by multiplex PCR. Mitogenicity of supernatants from each of these isolates was determined using peripheral blood leucocytes (PBL) from three healthy donors and assigned a grade, with +++ being highly mitogenic. *S. aureus* from 5 recurrent tonsillitis (RT) and 13 hyperplasia patients were profiled; each line is a representative isolate from an individual patient.

As *S. aureus* was common in tonsil tissues and was also cultured from 18 individuals, there were sufficient isolates available to determine the presence of *sag* genes and mitogenic activity. All isolates contained at least one *sag* gene but those containing *selx* (encoding staphylococcal enterotoxin-like toxin X) only were not mitogenic. Half of these isolates (9/18) were highly mitogenic (defined as ++ or +++ in proliferation assays) and contained one or more genes encoding for staphylococcal enterotoxins, typically *seb*, *sec,* or *seg* (Figure 2). All three GAS isolates were mitogenic in this assay (Table S1) and were tested for a limited panel of SAg (*speA*, *speC*, *speG,* and *smeZ*). The *emm28* strain was both highly mitogenic (+++) and positive for *speC*, *speG*, and *smeZ* whereas *speC* was not amplified from the two *emm89* isolates (mitogenicity = ++).

### 2.5. Potential for S. aureus to Interact with Immune Cells

**Figure 3:**
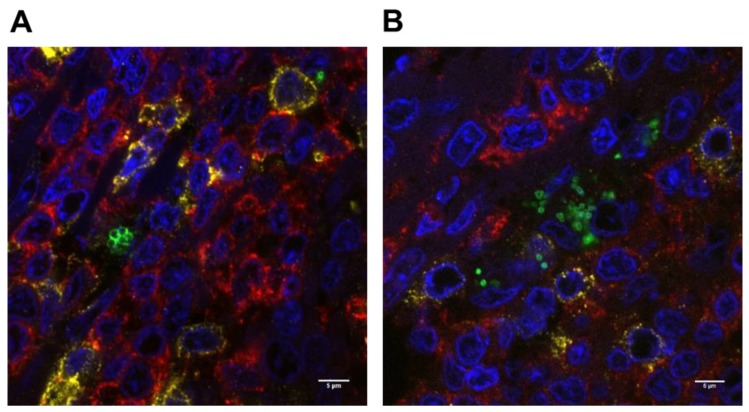
*S. aureus* and GAS are located in close proximity to B and T cells in the tonsils. Clusters of *S. aureus* (**A**) or GAS (**B**) detected by immuno-labelling and confocal microscopy in paraffin-embedded tonsillar tissue. Green = *S. aureus* or GAS; blue = DAPI; yellow = CD3^+^ T cells; red = CD20^+^ B cells. 100× magnification.

Three-color fluorescent antibody labeling was used to determine whether *S. aureus* was present in close proximity to immune cells in situ. An initial examination of hematoxylin- and eosin-stained tissue sections indicated that inflammatory cells, particularly neutrophils, were rare in tonsil tissues and not present in the vicinity of bacteria. In all instances, *S. aureus* was found in close proximity to CD3^+^ T cells and CD20^+^ B cells in the tonsil tissue (Figure 3A). GAS was also located within clusters of B and T cells (Figure 3B). It has been reported that *S. aureus* may sequester in an intracellular location [9], therefore sites where *S. aureus* appeared to be closely associated with immune cells were closely examined by confocal microscopy but this approach showed no conclusive evidence of intracellular localization of *S. aureus*.

### 2.6. Influence of S. aureus on Major Immune Cell Populations

*S. aureus* is common in tonsil tissue and closely associated with immune leucocytes, therefore potential differences in major immune cell subsets were examined by comparing the proportion of CD3^+^, CD4^+^, CD8^+^ T cells, or CD19^+^ B cells in tonsils and blood from patients that were culture positive or negative for tonsillar *S. aureus.* There were no significant differences in the proportion of these cell subsets in either the tonsils or peripheral blood in the presence of *S. aureus* (Supplemental materials, Figure S1). Similarly, the CD3^+^CD4^+^ T helper population had no changes in frequency of IL-2 receptor (CD25) or CXCR5 (TfH marker) expression (Supplemental materials, Figure S1). There were also no significant differences between OSA and RT patients (Supplemental materials, Figure S2).

**Figure 4:**
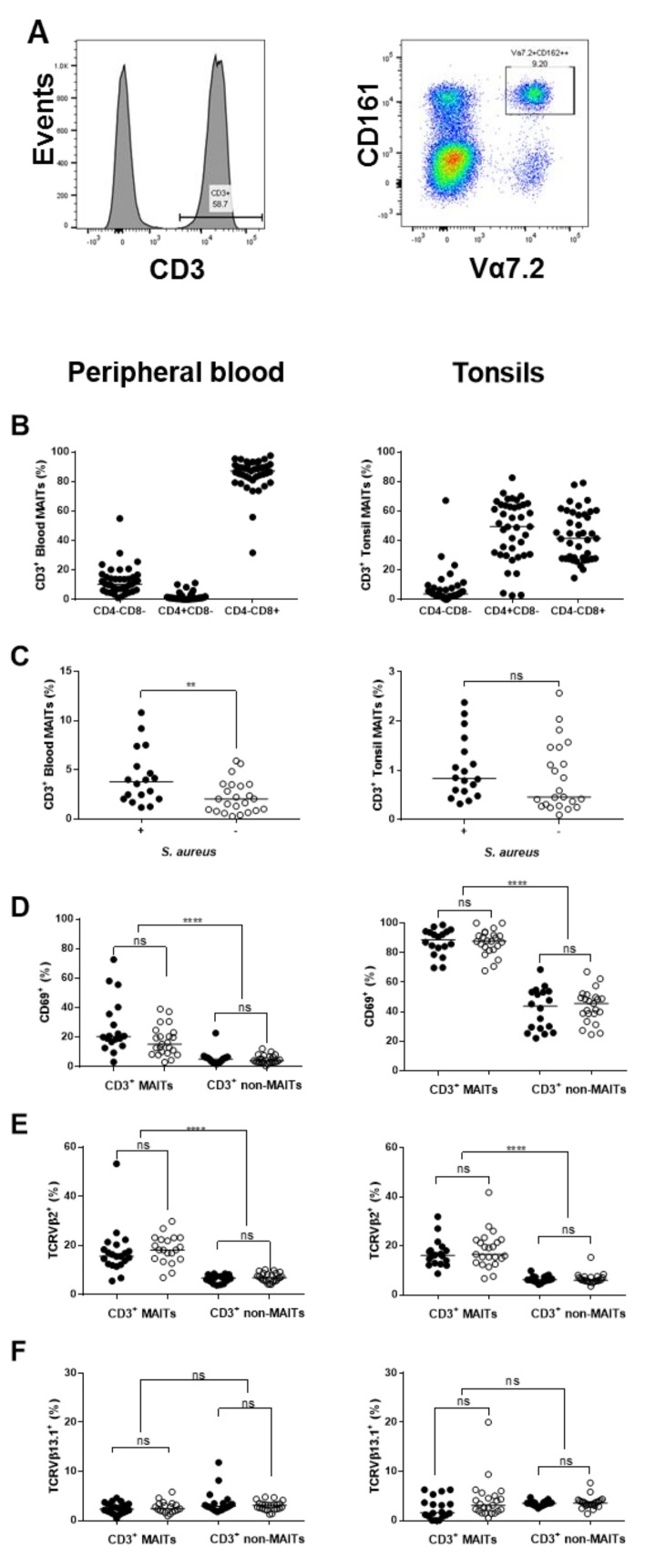
Characterization of mucosal-associated invariant T (MAIT) cells and the effect of *S. aureus* in the tonsils. A comparison of CD3^+^ MAIT and non-MAIT cells in blood and tonsil tissue in patients identified as culture positive (filled circle) or negative (open circle) for *S. aureus*. MAIT cells were identified as CD3^+^ CD161^++^Vα7.2^+^ (**A**) and the remaining CD3^+^ population classified as non-MAIT cells. These cell populations were quantified in blood or tonsil tissue (**B**) and then assessed for CD4 and CD8 (**C**), CD69 (**D**), TCR Vβ2 (**E**)**,** and TCR Vβ13.1 (**F**). Values are expressed as a percentage of CD3^+^ cells. Each point is a value from an individual patient and the horizontal line is the median. The two patient groups were compared with a two-tailed Mann–Whitney test or a Kruskal–Wallis test with Dunn’s multiple comparison test applied.

The possible impact of local exposure to *S. aureus* on CD3^+^CD161^++^Vα7.2^+^ mucosal-associated invariant T (MAIT) cells, which directly respond to metabolites produced by *S. aureus* [30] and are in return targeted by bacterial superantigens [28], was examined by flow cytometry (Figure 4A). It was noted that peripheral blood MAIT cells were predominantly CD8^+^ whereas tonsil tissue contained roughly equivalent proportions of CD4^+^ and CD8^+^ MAITs (Figure 4B). The median percentage of MAIT cells was increased in peripheral blood (3.8% vs. 2.0%, *p* = 0.006) and tonsil tissue (0.83% vs. 0.46%, ns) of *S. aureus* culture positive individuals (Figure 4C). Tonsillar MAIT cells were highly activated, with a median of 89% positive for the late activation marker CD69 compared to 20% of MAIT cells in blood, but *S. aureus* status had no impact on expression of CD69 in either tissue (Figure 4D). MAIT cells were significantly activated compared with non-MAIT cells from the same tissue (*p* < 0.0001) (Figure 4D). A common target of Staphylococcal and Streptococcal SAg, TCR Vβ2, is enriched in the MAIT cell population [36,37,38] therefore we determined the proportion of these cells in blood and tonsil tissue; TCR Vβ13.1 was also detected in these cells as a non-targeted control. The proportion of TCR Vβ2^+^ MAITs was comparable in both tissues (median = 16%), unaffected by the presence of *S. aureus* and significantly elevated compared to non-MAIT T cells (median = 6%, *p* < 0.0001) (Figure 4E). There was no difference in the proportion of TCR Vβ13.1^+^ MAITs relative to non-MAITs (median = 2–3%) and the proportion of TCR Vβ13.1^+^ MAITs was unchanged by *S. aureus* in either tissue (Figure 4F). A comparison of MAIT cells in RT or OSA patients indicated that tonsillar MAIT cells were significantly reduced in RT patients (median of 0.46% vs. 0.955%, *p* = 0.0306; Supplemental materials, Figure S3A) but no significant differences in activation status, TCR Vβ2 or TCR Vβ13.1 frequency, were detected (Supplemental materials, Figure S3B–D).

### 2.7. Assessment of TCR Vβ Profiles in Tonsils and Peripheral Blood

Tonsil TCR Vβ profiles were determined for all patients and skewing of TCR Vβ family members determined in comparison to reference values. Tonsil CD4^+^ and/or CD8^+^ TCR Vβ populations were skewed in 27 (65%) of participants, including 10 *S. aureus*-positive, 3 GAS-positive, and 11 *S. aureus*/GAS-negative individuals, with no association to RT or hyperplasia (Supplemental materials, Table S1). Skewing was more commonly detected in CD8^+^ T cells than CD4^+^ T cells. A significant increase in the proportion of both CD4^+^ and CD8^+^ T cells from the same TCR Vβ subset, which is more likely to be indicative of strong perturbation of TCR Vβ family members, was detected in only three individuals: An RT patient with GAS (*emm*28) and skewing of TCR Vβ2; another RT patient with skewing of TCR Vβ12; and a tonsillar hyperplasia patient with *S. aureus* and skewing of TCR Vβ13.2 (Supplemental materials, Table S1). 

**Figure 5:**
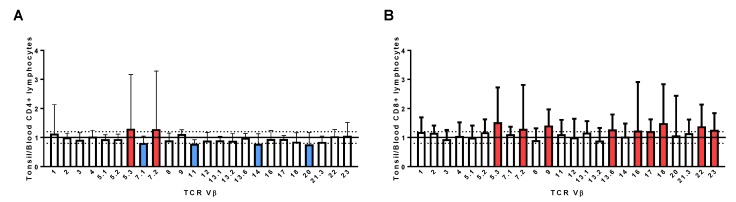
Tonsil TCR Vβ profiles normalized to peripheral blood for all patients in the study. Tonsil TCR Vβ profiles were normalized to peripheral blood profiles and data from all 41 patients combined to highlight any consistent differences for both CD4^+^ (**A**) and CD8^+^ (**B**) T cells; family members with a mean value of ≥1.2 are highlighted in red, those with a mean value of ≤0.8 are highlighted in blue. Data are mean + standard deviation.

Tonsil TCR Vβ values were normalized to peripheral blood leucocytes (PBL) to confirm that the TCR Vβ skewing observed was localized to the tonsil tissue and not due to individual variability in distribution of TCR Vβ family members. Data from all individuals were first combined to ascertain whether there were any consistent alterations in tonsil CD4^+^ or CD8^+^ TCR Vβ profiles relative to peripheral blood. Most CD4^+^ TCR Vβ family members had comparable values (1.0 +/− 0.2) to peripheral blood, but a small number of family members had mean values outside this range including Vβ5.3 (1.3), 7.2 (1.29), 14 (0.78), and 20 (0.763) (Figure 5A). Nine CD8^+^ TCR Vβ family members had mean values ≥1.2 relative to peripheral blood, including Vβ5.3 (1.52) and 7.2 (1.29) (Figure 5B) and none had mean values ≤0.8. Due to the variability of the CD8^+^ profiles, further analysis focused on CD4^+^ TCR Vβ profiles. A cut-off of ±0.5 was selected as the threshold for skewing of CD4^+^ TCR Vβ family members in individual patients. This analysis indicated that 29 (71%) individuals had an altered distribution of tonsil CD4^+^ TCR Vβ cells relative to peripheral blood (Table S1) and was more common in patients with RT (20/23) than those with hyperplasia (10/18) but was not associated with the presence of *S. aureus*. 

**Figure 6:**
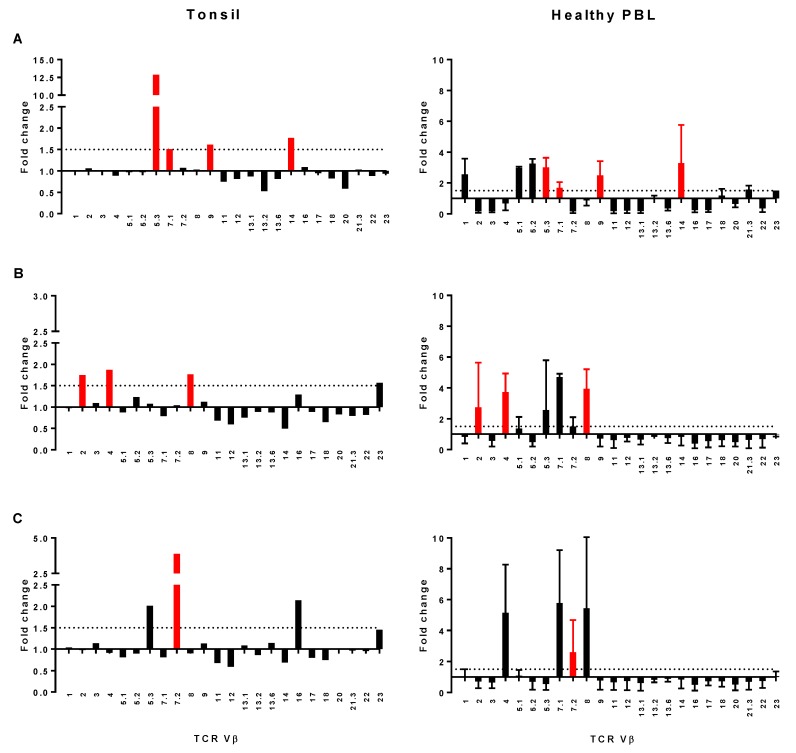
Comparison of TCR Vβ profiles from tonsils and healthy PBL stimulated with bacterial supernatants. Individual profiles showing strong localized TCR Vβ skewing in the tonsil (LHS) aligned with TCR Vβ profiles of healthy PBL after stimulation with culture supernatant from *S. aureus* or GAS isolated from these individuals (RHS); red bars identify TCR Vβ family members expanded in both sets of cells. Results are from three patients who underwent surgery to treat RT who were positive for *S. aureus* (**A**), GAS *emm28* (**B**)**,** or GAS *emm89* (**C**) respectively. Tonsil data are normalized to peripheral blood profiles from the same individual. PBL data are shown as fold change relative to unstimulated PBL, incorporating mean values plus standard deviation from two healthy donors.

The most striking examples of skewing were from three RT patients with altered distributions of selected CD4^+^ TCR Vβ family members relative to both peripheral blood (Figure 6A–C) and reference values (Supplemental materials, Table S1). Culture supernatants from the bacteria isolated from these patients were used to stimulate PBL to determine whether a comparable TCR Vβ profile could be reproduced in vitro (Figure 6A–C). A patient with mitogenic *S. aureus* containing *sell*, *selx,* and the *egc* genes had strong skewing of TCR Vβ5.3, 7.1, 9, and 14 in tonsil tissue; these family members were also expanded in PBL exposed to supernatant from this *S. aureus* isolate (Figure 6A). The other individuals were culture positive for mitogenic GAS *emm*28 and *emm*89 isolates, with skewing of TCR Vβ 2, 4, 8 (Figure 6B) or TCR Vβ5.3, 7.2, and 16 respectively (Figure 6C). Supernatant from the GAS *emm*28 isolate stimulated selective expansion of TCR Vβ 2, 4, and 8 (Figure 6B) in healthy PBL, whereas the profile from the *emm*89 strain showed less overlap, with expansion of TCR Vβ7.2 but also TCR Vβ4, 7.1, and 8 (Figure 6C).

## 3. Discussion

This study builds on an earlier finding that skewing of TCR Vβ subsets is relatively common in tonsillar tissue [24], demonstrating that selective expansion of TCR Vβ populations is localized to the tonsils and that it is possible to partially reproduce patient TCR Vβ profiles in vitro using supernatants from their own mitogenic *S. aureus* or GAS strains. Tonsillar hyperplasia is a multifactorial disease and there is now evidence to suggest that chronic exposure of immune cells to SAg producing bacteria may contribute to this condition, particularly RT, in a subset of patients.

Many of the *S. aureus* strains isolated from this patient cohort contained genes for potent SAgs and were highly mitogenic in in vitro assays. We previously found that patients with tonsillar *S. aureus* and RT were more likely to present with skewing of their tonsil TCR Vβ subsets [24]. This association was not apparent in this set of patients and illustrates the challenges associated with obtaining conclusive evidence from a small cohort of individuals, with RT or OSA resulting from a variety of causes. Nonetheless, several RT patients had striking examples of skewed tonsillar CD4^+^TCR Vβ family members relative to peripheral blood that were strongly suggestive of local SAg exposure. Supernatants from the patient’s mitogenic *S. aureus* or GAS isolates drove selective expansion of some of these TCR Vβ family members. An *S. aureus* isolate containing *sell* and *selx* genes and the *enterotoxin gene cluster (egc)* stimulated expansion of TCR Vβ5.3, 7.1, 9, and 14 in both tonsil tissue and PBL, which is consistent with the activities of SAgs encoded by the *egc* [39]. An individual infected with a GAS *emm*28 strain positive for *speC*, *speG,* and *smeZ* had expansion of tonsillar CD4+ TCR Vβ2, 4, and 8 T cells suggestive of Streptococcal Mitogenic Exotoxin Z (SMEZ) [40,41]. A similar profile was produced in vitro after stimulation of PBL with supernatant from this isolate. Tonsil and PBL profiles from a patient carrying an *speG*+, *smeZ*+, and GAS *emm*89 isolate were not as informative: the tonsil TCR Vβ profiles (expansion of Vβ 5.3 and 7.2) do not align with known Vβ targets of streptococcal SAgs [14]. In vitro stimulation of PBL with bacterial supernatant also showed stimulation of Vβ 7.2, suggesting the presence of a secreted molecule capable of targeting this family member, but also expansion of Vβ4 and 8, which can be attributed to the presence of *smeZ*.

A global analysis of all patient tonsil CD4+ TCR Vβ profiles demonstrated that expansion of TCR Vβ5.3 and TCR Vβ7.2 was most variable across this cohort and frequently expanded relative to PBL. These family members are not known targets of Streptococcal Sags [14], whereas TCR Vβ5.3 is targeted by multiple Staphylococcal SAgs including members encoded in the *egc* frequently carried by *S. aureus* [42] as well as SEA, SED, and SElL; and TCR Vβ7.2 by SEA [39]. Conversely, a further four TCR Vβ family members were under-represented in the tonsils, including additional targets of Staphylococcal SAgs: TCR Vβ7.1 (SEIL), Vβ11 (SER), Vβ14 (SEB, SEC, SEG, SER, SElU), and Vβ20 (SEB, SEC) [14,39]. *S. aureus* with genes encoding for most of these *sag* genes were cultured from tonsil tissue and the *egc* was present in 50% of these isolates. These TCR Vβ profiles are suggestive of local exposure to SAg commonly produced by *S. aureus*, however it is difficult to make a direct link between Sag-producing bacteria currently resident in the tonsils and alterations in TCR Vβ populations. Exposure to SAg leaves a lingering footprint on the T cell repertoire and profiles may be attributable to past infections or exposure to multiple SAgs. 

Acquiring stronger evidence of SAg activity in the tonsils is likely to require a combination of very careful selection of specific subsets of patients along with sophisticated immune profiling technologies. Specifically, data on patient HLA class II types could provide important linkages with SAg susceptibility, but collecting this information was beyond the scope of this study. A recent analysis of tissues from GAS RT patients has a compelling explanation as to why only a subset of the many individuals exposed to GAS actually go on to develop GAS RT. Tonsil tissues from GAS RT patients were observed to have a lower frequency of TfH, smaller germinal centers, and a reduced circulating IgG response to SpeA, a common SAg produced by GAS [34]. Vulnerability to recurrent infections was then linked to an HLA class II-linked susceptibility to SpeA [34]. Direct exposure of tonsillar cells to SpeA has also revealed that SpeA targets TfH cells to impair B cell function, leading to a reduction in antibody production [33]. However, our study found no reduction of TfH in tonsils from RT patients, probably because our RT cohorts were not specifically selected for multiple episodes of GAS RT. CXCR5 was also the sole marker used to identify CD3^+^CD4^+^ TfH, rather than a more extended phenotyping panel. RT is a complex disease and a focus on links to other SAgs may reveal additional genetic susceptibilities and in vivo functions of these key virulence factors.

*S. aureus* is a frequent colonizer of tonsillar tissue and located in close proximity to key immune cells. Clusters of bacteria were ensconced within tonsil tissue with no evidence of phagocytic cells, such as neutrophils, in their vicinity. There was also no indication that *S. aureus* status impacted on the frequency or activation status of major B and T cell subsets in the tonsils. The possible in vivo consequences of *S. aureus* exposure on MAIT cells, a recently identified subset of T cells of particular interest in relation to both *S. aureus* and GAS, has not previously been determined. In our study, MAIT cells typically comprised <1% of CD3^+^ T cells in the tonsils and were significantly activated compared to those in circulation, in line with previous reports on tissue resident MAIT cells from the liver [43] and the gut [44], suggesting the tonsils are a stimulatory environment for these cells. Blood and tonsillar MAITs were elevated in individuals that were culture positive for tonsillar *S. aureus*. In contrast, MAIT cells were significantly reduced in tonsil tissues, but not blood, from RT patients. Circulating MAIT cell populations initially decline, then rebound to elevated levels several weeks after a controlled acute infection with *Salmonella* paratyphi A [45], whereas the frequency of these cells is decreased in patients with severe sepsis [46] or in response to chronic bacterial infections [30,47,48]. Continuous exposure to selected bacterial metabolites may sustain a constant population of activated MAIT cells in the tonsils to maintain immune surveillance in the nasopharynx and peripheral sites. Conversely, it was recently demonstrated that MAIT cells are highly responsive to Staphylococcal Enterotoxin B (SEB), rapidly produce large quantities of pro-inflammatory cytokines, and become anergic [28]. Mitogenic isolates of *S. aureus* containing the genes for several potent SAgs including *sea*, *seb*, *sec,* and *tst-1* are relatively common in the tonsils, both in this study cohort and a previous set of patients [24], suggesting the potential for MAIT cells to be exposed to and disarmed by locally produced SAgs. Selected TCR Vβ family members are over-represented in MAIT cells including TCR Vβ2, which is targeted by several potent SAg from both *S. aureus* and GAS, including Toxic Shock Syndrome Toxin-1, SpeC, and SMEZ [14]. In our patient cohort, the proportion of TCR Vβ2^+^ MAIT cells were not significantly different in the tonsils compared to peripheral blood of *S. aureus*-infected individuals, suggesting no targeted expansion or deletion of this cell type. It has been reported that TCR Vβ13.2^+^ MAIT cells are highly susceptible to the effects of SEB, however this particular family member was not examined in our study. Production of SAgs may be an important mechanism used by both *S. aureus* and GAS to disable the local immune response and remain resident in mucosal tissues. 

This study shows several strong examples of tonsillar TCR Vβ skewing in conjunction with the presence of mitogenic strains of *S. aureus* or GAS capable of eliciting similar TCR Vβ skewing profiles, suggesting that tonsil immune cells are exposed to these potent toxins. SAg may play a role in driving tonsillar hyperplasia in some individuals by disabling or subverting the local tonsil T cell response, including innate lymphoid cells such as MAIT cells, to facilitate ongoing susceptibility to infection. Both *S. aureus* and GAS were confirmed to be located within tonsil tissue in sites containing immune cells, therefore any SAgs produced by these bacteria are highly likely to encounter receptive populations of cells. However, in most instances, it was difficult to align the TCR Vβ profiles detected in the tonsils with the SAg profile of the colonizing strain, if present. Addressing these issues will require testing the effect of multiple SAgs on human PBMC in vitro, ideally combined with humanized animal models to permit an in-depth examination of issues such as the impact of isolates expressing multiple potent SAgs on the TCR Vβ repertoire, whether exposure to Sag-producing bacteria directly affects MAIT cell responses, and if prior exposure to a highly mitogenic isolate results in enhanced susceptibility to future infections. These approaches, combined with recent advances in immune cell phenotyping and genetic analyses, offer many opportunities to advance our knowledge of the drivers of tonsillar conditions.

At present, penicillin-based antibiotics are commonly prescribed for tonsillitis, which effectively targets GAS, but is unlikely to inhibit growth of *S. aureus*. Children with OSA are also regularly prescribed antibiotics and usage was comparable between the RT and OSA cohorts in this study. Inappropriate treatment with antibiotics risks the creation of new niches that permit overgrowth of *S. aureus* or other pathogens in this site, potentially exacerbating these conditions. Antibiotics are also likely to be only transiently effective in individuals with an HLA-linked susceptibility to recurrent GAS infection [34]. The drivers of tonsillar hyperplasia are multifactorial, but a better understanding of the role of SAg-producing bacteria in the tonsils may lead to improvements in non-surgical approaches for treating tonsillar hyperplasia, either in the form of a vaccine or application of more effective antibiotics.

## 4. Materials and Methods

### 4.1. Patients

Tonsil tissue, a tonsil swab, and a blood sample were obtained from patients undergoing a routine tonsillectomy for RT or tonsillar hyperplasia at Gillies Hospital, Auckland, New Zealand. Approval to collect these specimens was gained from The University of Auckland Human Ethics Committee (ref: 010200). Written informed consent was acquired from the donors or their guardians prior to surgery. Patient age, gender, and the reason for surgery were collected and anonymized. Details of recent antibiotic usage was obtained and combined with specific prescribing information sourced from Auckland District Health Board databases. 

### 4.2. Bacteriology

Tonsil specimens were sampled for bacterial growth, including detection of *S. aureus* and GAS as previously described [24]. Briefly, tissues were homogenized, duplicate samples cultured overnight at 37 °C with/out 5% CO_2_ on Columbia Blood Agar (CBA; Fort Richard, Auckland, New Zealand), and the total number of bacteria present after growth in aerobic conditions determined. Putative *S. aureus* colonies were cultured overnight on Mannitol Salt Agar (MSA; Fort Richard), and MSA positive colonies confirmed as *S. aureus* on the basis of catalase production and *S. aureus*-selective PCR [24,49]. GAS isolates were identified as β-hemolytic on CBA then confirmed using an established *emm*-typing PCR [50] and the product sequenced to identify the *emm* type. 

### 4.3. Histology

Tissue pieces from both tonsils were placed in Carnoy’s Fixative (60% *v*/*v* Ethyl Alcohol, 30% *v*/*v* Chloroform, 10% *v*/*v* Acetic Acid) for ≥24 hours, embedded in paraffin and cut into 4 µm sections. Sequential tissue sections were stained with hematoxylin and eosin to examine tissue architecture or antibody labelled for fluorescence microscopy. Tonsil sections were stained with antibodies to CD3, CD20, and *S. aureus* or GAS in conjunction with the nuclear marker DAPI (Invitrogen). The primary and secondary antibodies used are detailed in Table 3. Tissues were de-waxed followed by heat-induced epitope retrieval in the 2100 Retriever system (PickCell laboratories, Amsterdam, The Netherlands) in either 10 mM citric acid, pH 6 for detection of *S. aureus,* or EDTA-Tris buffer (10 mM Tris, 1.3 mM EDTA, pH 8.5) for GAS. During the labelling process, the tissues were blocked in 10% *v*/*v* normal goat serum for 30 minutes; treated with antibodies diluted in Novocastra IHC diluent (Leica Biosystems, Wetzlar, Germany) added overnight at 5 °C (primary antibodies) or for 1 hour at room temperature (secondary antibodies); and washed in Tris buffered saline (24.8 mM Tris, 0.137 mM NaCl, 2.7 mM KCl) between labelling steps. DAPI was included at a dilution of 1:10000 during the secondary antibody incubation step. Fully stained tissues were mounted in anti-fade mountant (Citifluor, Hatfield, PA, USA) and examined by fluorescence microscopy. Negative controls were included for every piece of tissue examined to verify specificity of the labeling. A positive control of tonsil containing either *S. aureus* or GAS was included with each batch of stains. Sections of both tonsils were screened for bacteria, with an average total area of 104.5 mm^2^ (range: 40.2–182 mm^2^) scanned for each patient. All tissue sections were screened for the presence of *S. aureus* or GAS, and images of bacteria from each positive individual were retained for confirmation by an experienced histologist (SW). Regions containing bacteria were assessed for the presence of T and B leucocytes across multiple patients and composite images were recorded. 

### 4.4. Superantigen Profiling

All *S. aureus* isolates were *sag* profiled for Staphylococcal Enterotoxins (SE) and Staphylococcal Enterotoxin-like (SEl) genes including members of the *enterotoxin gene cluster* (*egc*) by multiplex PCR as previously described [24,51]. GAS isolates were processed as per the *emm* typing PCR and tested for the presence of *speA*, *speC*, *speG,* and *smeZ* using previously published primer sets and conditions [52]. Diluted supernatants from overnight cultures of all isolates were tested for mitogenicity on human peripheral blood leucocytes (PBL) from at least three healthy consenting donors [53]. Supernatants from GAS grown overnight in RPMI 1640 supplemented with 10% *v*/*v* fetal bovine serum (Gibco by Life Technologies, Auckland, New Zealand) were also tested. PBL were cultured for 72 hours in the presence of serial 10-fold dilutions of *S. aureus* or GAS supernatants with a starting dilution of 1:40, and proliferation quantified by measuring the uptake of 0.25 µCi/well [methyl-^3^H] thymidine (Perkin-Elmer, Waltham, MA, USA) during the final 6 hours of the incubation period. The degree of mitogenicity was defined as the reciprocal of the dilution that stimulated proliferation >3 times background. Supernatants that stimulated proliferation at a dilution of ≥400,000 were recorded as ‘+++’; 4000 – 40,000 as ‘++’; 40 – 400 as ‘+’; and ≤40 as ‘−’ [24]. 

### 4.5. Flow Cytometry

Tonsil tissue was stored on wet ice in phosphate buffered saline (PBS) and transported to the laboratory for processing within 2 hours of surgery. Tonsil tissues were minced in PBS, further dissociated using a 70 µm cell strainer, and leucocytes isolated by density gradient centrifugation over Ficoll-Hypaque™ PLUS (GE Healthcare, Uppsala, Sweden) [54]. Peripheral blood samples were collected into heparin tubes and diluted 1:1 in PBS prior to centrifugation over Ficoll-Hypaque at 400 *g* for 30 minutes. Isolated leucocytes were washed twice in PBS, enumerated using a hemocytometer, and up to 10^6^ cells/tube used for antibody labeling for flow cytometry. Cells were re-suspended and washed in FACS Buffer (2% *v*/*v* fetal bovine serum, 0.02% *v*/*v* sodium azide in PBS) for all subsequent steps then suspended in FACS Buffer supplemented with 0.5 µg/mL propidium iodide (Invitrogen, Eugene, OR, USA) for data acquisition. Specific antibody details and combinations are detailed in Table 3. Tonsil and peripheral blood leucocyte TCR Vβ profiles were determined using antibodies to CD4 and CD8 in conjunction with the IOTest^®^ Beta Mark TCR Vβ Repertoire Kit (Immunotech, Marseille, France) as per the manufacturer’s instructions. At least 5000 viable CD8^+^ (tonsil) or CD4+ (blood) events were collected per sample. Major leucocyte subsets (CD3^+^, CD4^+^, CD8^+^, and CD19^+^) were identified by positive cell surface marker staining and expressed as a proportion of viable WBC. CD4^+^ T helper cell subsets were further characterized as CD3^+^CD4^+^CD25^+^ or CD3^+^CD4^+^CXCR5^+^ TfH [55,56]. B cells were identified based on presence of CD19, with maturity and activation status determined with antibodies to CD21 and CD27, respectively. MAIT cells were identified as CD3^+^ CD161^++^Vα7.2^+^ [57,58] with additional characterization using antibodies to CD4, CD8, CD69, TCR Vβ2, or TCR Vβ13.1. All flow cytometry data were acquired on a BD LSR II with FACS Diva Software v6.1.1 (BD Biosciences, Franklin Lakes, NJ, USA) and analyzed with FlowJo software v7.6.5 (Tree Star Inc, Ashland, OR, USA). Fluorescence-minus-one controls were used to determine placement of gates. Single, viable cells were selected on FSC/SSC profiles and the exclusion of PI. Proportionate representation of CD4^+^ or CD8^+^ TCR Vβ populations was determined and TCR Vβ skewing defined as values > two standard deviations above control values supplied with the TCR Vβ repertoire kit [59]. Alternatively, tonsil TCR Vβ values were normalized to blood TCR Vβ values to examine possible differences between local versus peripheral TCR Vβ profiles.

In-depth TCR Vβ profiling was also conducted on PBL from two healthy donors after 6 days of in vitro stimulation with 1% *v*/*v* bacterial culture supernatant, with the addition of 50 IU/mL IL-2 (PeproTech Asia, Rehovot, Israel) after 72 hours [40]. Data are presented as the fold difference between PBL stimulated with bacterial culture supernatants and those in medium alone.

### 4.6. Statistical Analyses

Statistical analyses and plots were produced in Prism 7 (GraphPad Software, San Diego, CA, USA). Variables including tonsil weight, leucocyte count, and CFU of bacteria were compared for RT and OSA groups by Mann–Whitney test. The impact of *S. aureus* on major immune populations was analyzed by Mann–Whitney test or unpaired t-test for datasets containing non-parametric or parametric data, respectively. A comparison of characteristics of MAIT and non-MAIT CD3^+^ T cells were performed with a two-tailed Wilcoxon matched-pairs signed rank test. All plots include individual data points from each patient and the median value. A *p* value of <0.05 was used as the cut-off for statistical significance.

## Figures and Tables

**Table 1:** Patient information including tonsil weights, leucocyte counts, and bacterial load.

Parameter Median *(Range)*	Recurrent Tonsillitis (n = 23)	Tonsillar Hyperplasia (n = 18)	Combined (n = 41)
Male/Female	12/11	9/9	21/20
Age (years)	4 *(1–42)*	4 *(2–58)*	4 *(1–58)*
Tonsil Weight (g)	5.42 *(2.31–16.24)*	5.92 *(4.37–8.87)*	5.74 *(2.31–16.24)*
Tonsil leucocyte/g × 10^6^	193 *(60–363)*	264 *(109–410)*	223 *(60–410)*
Tonsil CFU/g × 10^6^	0.99 *(0.055–4.5)*	1.3 *(0.08–5.46)*	1.16 *(0.055–5.46)*
Swab CFU × 10^6^	1.4 *(0.01–2.3)*	0.99 *(0.11–2.1)*	1.1 *(0.01–2.3)*

**Table 2:** Detection of *S. aureus* and Group A *Streptococus* (GAS) in tonsils from 41 patients by culture and immuno-histology.

Pathogen	Culture				Immuno-Histology	Combined (%)
	Surface Only	Tissue Only	Surface & Tissue	Total		
*S. aureus*	0	8	10	18	15	25 (61)
GAS	0	2	1	3	3	3 (7)
GGS ^1^	0	3	1	4	n.d.	4 (10)
GCS ^2^	0	0	1	1	n.d.	1 (2.5)

^1^ Group G Streptococcus; ^2^ Group C Streptococcus.

**Table 3:** Details of the antibodies used in this study.

Specificity	Fluorochrome	Clone	Quantity or Dilution Used ^1^	Source
CD3	Unconjugated ^2^	Polyclonal (rabbit)	1:600	Cell Marque
CD3	Unconjugated ^3^	F7.2.38 (mouse IgG1)	1:300	Dako
CD3	FITC ^5,6,7^	UCHT1	2.5 µL	BD Biosciences
CD4	APC-Cy7 ^4,5,6^	RPA-T4	2.5 µL	BD Biosciences
CD8	AF-647 ^4,6^ or BUV395 ^5^	RPA-T8	2.5 µL	BD Biosciences
CD19	PE ^7^	HIB19	20 µL	BD Biosciences
CD20	Unconjugated ^2,3^	L26 (mouse IgG2a)	1:100	Leica
CD21	APC ^7^	B-ly4	5 µL	BD Biosciences
CD25	PE ^6^	M-A251	20 µL	BD Biosciences
CD27	PE-CF594 ^6,7^	M-T271	1 µL	BD Biosciences
CD69	PE ^5^	FN50	20 µL	BD Biosciences
CD161	APC ^5^	DX12	20 µL	BD Biosciences
CXCR5	AF-647 ^6^	RF8B2	1 µL	BD Biosciences
Vα7.2	Pe-Cy7 ^5^	3C10	2.5 µL	BioLegend
Vβ2	PE ^5^	MPB2D5	20 µL	Immunotech
Vβ13.1	PE ^5^	IMMU 222	20 µL	Immunotech
*S. aureus*	Unconjugated ^2^	11-248.2 (mouse IgM)	1:400	Merck, MAB930
*S. pyogenes*	Unconjugated ^3^	Polyclonal (rabbit)	1:7000	Biorbyt orb99012
Mouse IgG1	AF-647 ^3^	Polyclonal (goat)	5 µg/mL	Thermo Fisher Scientific
Mouse IgG2a	AF-594 ^2,3^	Polyclonal (goat)	5 µg/mL	Thermo Fisher Scientific
Mouse IgM	AF-488 ^2^	Polyclonal (goat)	4 µg/mL	Thermo Fisher Scientific
Rabbit IgG	AF-488 ^3^ or AF-647 ^2^	Polyclonal (goat)	4 µg/mL or 5 µg/mL	Thermo Fisher Scientific

^1^ Quantity per 10^6^ cells for flow cytometry or dilution per slide for immunohistochemistry; ^2^ *S. aureus* triple label; ^3^ *S. pyogenes* triple label; ^4^ TCR Vβ profiling panel; ^5^ MAIT cell panel; ^6^ T cell activation panel; ^7^ B cell activation panel.

## Supplementary Materials

The following are available online at https://www.mdpi.com/2076-0817/8/3/90/s1, Figure S1: Presence of *S. aureus* in tonsil tissue does not alter major T and B cell subsets; Figure S2: Major T and B cell subsets are comparable in RT and OSA patients; Figure S3: Characterization of MAIT cells in RT and OSA patients; Table S1: TCR Vβ profiles from tonsil CD4^+^ or CD8^+^ T cells of RT or OSA patients relative to healthy reference values or normalised against peripheral blood.
