# Supplementary material for: Impact of Superantigen-Producing Bacteria on T Cells from Tonsillar Hyperplasia
Source: Pathogens. 2019 Jun 27;8(3):90. doi: 10.3390/pathogens8030090 (PMC6789895; doi:10.3390/pathogens8030090)
Supplement: Supplementary file 1 [file pathogens-08-00090-s001.pdf]

Table S1. TCR Vβ profiles from tonsil $CD4^+$ or $CD8^+$ T cells of RT or OSA patients relative to healthy reference values or normalised against peripheral blood. Each line represents the profile from an individual patient.

**Pathogen**[1]	**TCR Vβ skewing versus reference values**		**Staphylococcal Superantigen**		**$CD4^+$ TCR Vβ skewing tonsil/blood (fold change)**	
	CD4+	CD8+	Gene/Gene Cluster	Mito-genicity	≥1.5	≤0.5
RT						
S. aureus	-	-	*selx*	-	-	-
	-	-	*seb*	+++	-	16, 20
	-	5.3	*selp, selx*	+++	1	-
	-	-	*selx*	-	13.2	20
[3]	7.1, 14	18	*seg, sell, selx, egc*	+	5.3, 7.1, 9, 14	-
GAS *(emm28)*[3]	2, 4	2, 18	*speC, speG, smeZ*	+++	2, 4, 8, 23	14
GAS *(emm89)*[3]	16	18	*speG, smeZ*	++	5.3, 7.2, 16	-
GAS *(emm89)*	-	16, 18, 22, 23	*speG, smeZ*	++	-	13.2
StG62647	3, 4, 23	-	n/a	n.d.	14, 20	-
	-	-	n/a	n/a	-	18, 23
	2	16	n/a	n/a	22	18
	-	-	n/a	n/a	-	7.2, 20
	-	5.2, 5.3, 13.1, 18	n/a	n/a	23	1
	-	17	n/a	n/a	-	-
	-	-	n/a	n/a	5.3	23
	-	1	n/a	n/a	-	7.2
	-	18, 23	n/a	n/a	-	7.1, 20
	2	-	n/a	n/a	-	20
	-	-	n/a	n/a	-	18, 20
	-	5.1	n/a	n/a	-	-
	-	-	n/a	n/a	7.2, 13.2, 23	-
	-	11, 13.1, 23	n/a	n/a	18, 23	-
	12	12, 13.1	n/a	n/a	-	7.2
Hyperplasia						
S. aureus	-	-	*selx*	-	-	-
	-	1, 5.3, 16	*sec, seg, sell, selx, egc*	+++	14, 18	7.1
	-	22	*sed, seg, selx, egc*	+++	-	7.1, 7.2
	8	-	*seg, selx, egc*	+++	-	-
	-	-	*seg, selx, egc*	+	-	-
	-	-	*selx*	-	-	-
	-	-	*sea, seg, selx, tsst-1*	-	-	-
	2, 13.2	5.3, 13.2, 18, 23	*sec, sell, selx, egc*	+++	12	23
	-	7.1, 18	*selp, selx*	-	-	18, 23
	-	-	*seg, selx, egc*	++	-	-
S. aureus & StG245	23	-	*sec, seg, sell, selx, egc*	++	23	3, 7.2, 12, 14, 16, 20, 21.3

S. aureus & StC47A	2, 5.1	-	*selp, selx*	+	23	-
S. aureus & StG62647	-	1	*seb, sec*	+++	-	-
Stg6792	-	18, 22, 23	n/a	n/a	-	-
	-	-	n/a	n/a	20	-
	-	9	n/a	n/a	-	-
	-	5.3	n/a	n/a	-	7.2, 14, 20
	2	-	n/a	n/a	7.2	-

[1]Detected by culture ; [2]Skewing of the same TCR Vβ family members across CD4+ and CD8+ T cells and/or also increased relative to peripheral blood values are highlighted in bold font; [3]Isolates used for additional TCR Vβ profiling; n/a = not applicable

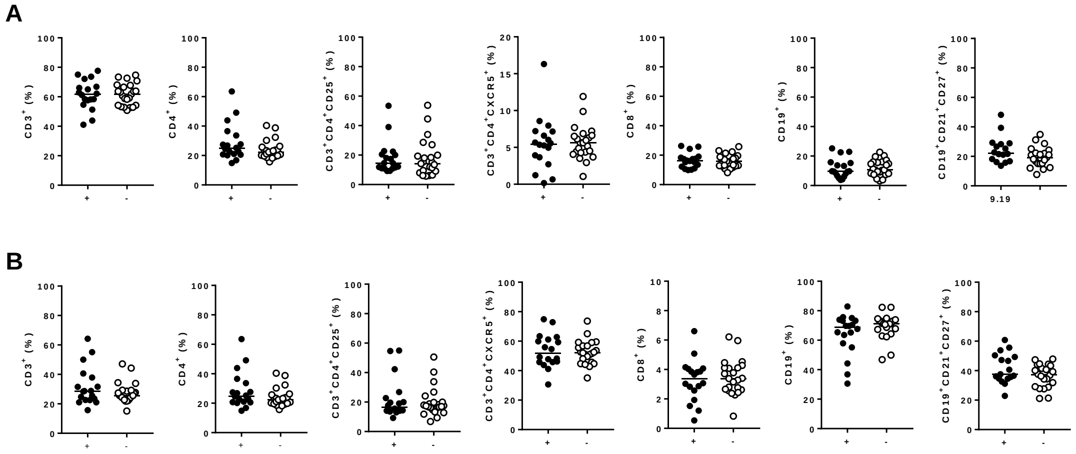

Figure S1. Presence of *S. aureus* in tonsil tissue does not alter major T and B cell subsets. Profiling of major immune cell subsets in peripheral blood **(A)** and tonsil tissue **(B)** in patients identified as culture positive (+) or negative (-) for *S. aureus*. Each point is a value from an individual patient and the horizontal line is the median. The two patient groups were compared with a two-tailed Mann-Whitney test or unpaired t-test.

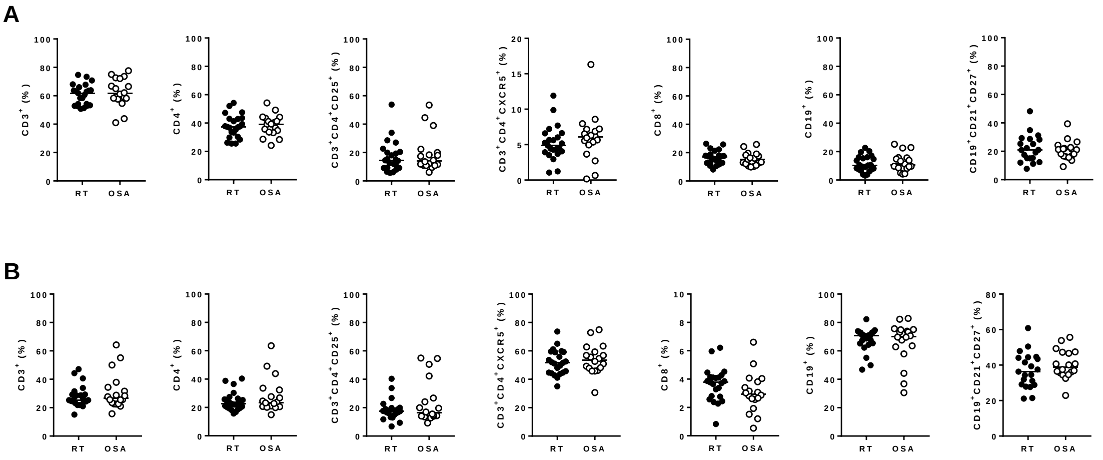

Figure S2. Major T and B cell subsets are comparable in RT and OSA patients. Profiling of major immune cell subsets in peripheral blood **(A)** and tonsil tissue **(B)** in RT and OSA patients. Each point is a value from an individual patient and the horizontal line is the median. The two patient groups were compared with a two-tailed Mann-Whitney test or unpaired t-test.

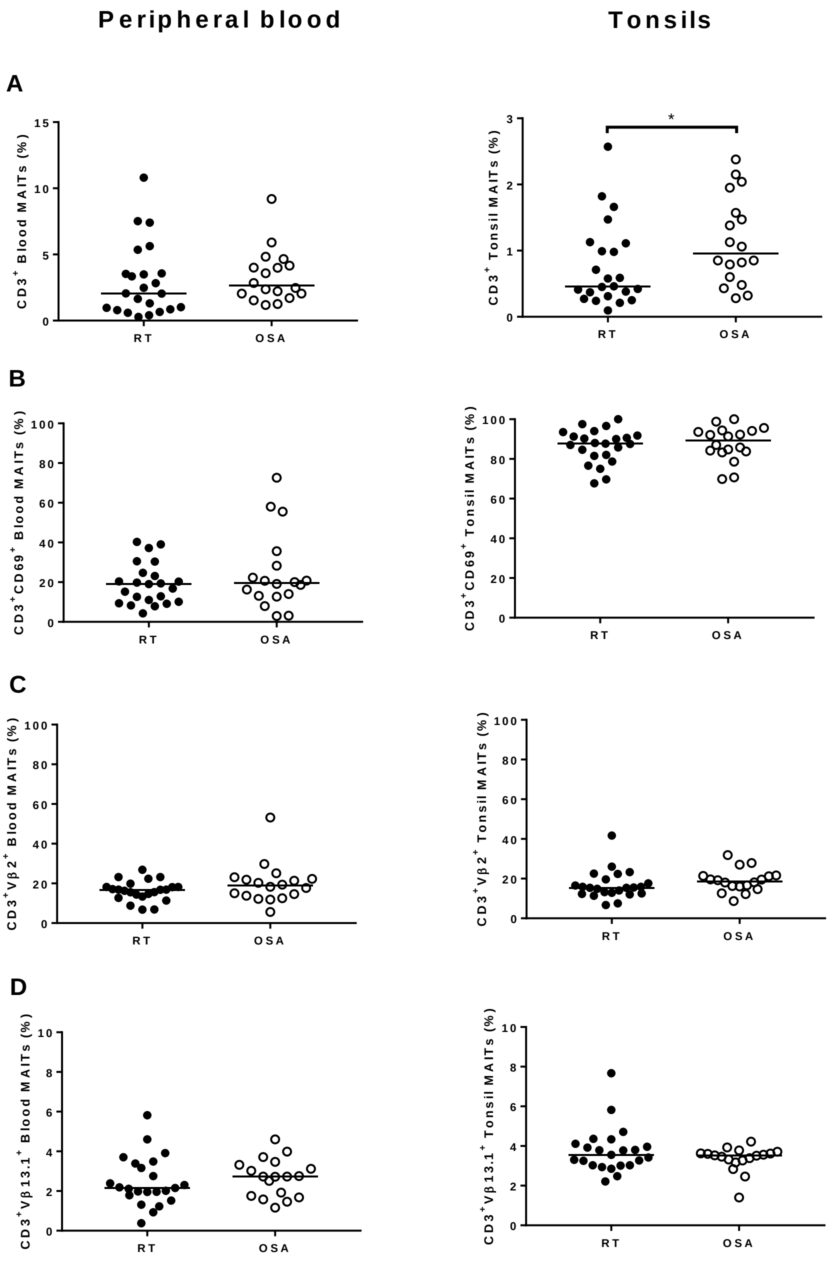

Figure S3. Characterisation of MAIT cells in RT and OSA patients. A comparison of CD3$^+$ CD161^{++}Vα7.2$^+$ MAIT in patients with RT or OSA. These cell populations were quantified in blood or tonsil tissue **(A)** and then assessed for expression of CD69 **(B)**, TCR Vβ2 **(D)** and TCR Vβ13.1 **(D)**. Values are expressed as a percentage of CD3$^+$ cells. Each point is a value from an individual patient and the horizontal line is the median. The two patient groups were compared with a two-tailed Mann-Whitney test.
